# Fhl1p protein, a positive transcription factor in *Pichia pastoris*, enhances the expression of recombinant proteins

**DOI:** 10.1186/s12934-019-1256-0

**Published:** 2019-11-29

**Authors:** Xueyun Zheng, Yimin Zhang, Xinying Zhang, Cheng Li, Xiaoxiao Liu, Ying Lin, Shuli Liang

**Affiliations:** 1 Guangdong Provincial Key Laboratory of Fermentation and Enzyme Engineering, South China University of Technology, Guangzhou, 510006 People’s Republic of China; 2 Guangdong Research Center of Industrial Enzyme and Green Manufacturing Technology, School of Biology and Biological Engineering, South China University of Technology, Guangzhou, 510006 People’s Republic of China

**Keywords:** *Pichia pastoris*, Fhl1p, Transcription factor, Enhanced protein production, Translation

## Abstract

**Background:**

The methylotrophic yeast *Pichia pastoris* is well-known for the production of a broad spectrum of functional types of heterologous proteins including enzymes, antigens, engineered antibody fragments, and next gen protein scaffolds and many transcription factors are utilized to address the burden caused by the high expression of heterologous proteins. In this article, a novel *P. pastoris* transcription factor currently annotated as Fhl1p, an activator of ribosome biosynthesis processing, was investigated for promoting the expression of the recombinant proteins.

**Results:**

The function of Fhl1p of *P. pastoris* for improving the expression of recombinant proteins was verified in strains expressing phytase, pectinase and mRFP, showing that the productivity was increased by 20–35%. RNA-Seq was used to study the Fhl1p regulation mechanism in detail, confirming Fhl1p involved in the regulation of rRNA processing genes, ribosomal small/large subunit biogenesis genes, Golgi vesicle transport genes, etc., which contributed to boosting the expression of foreign proteins. The overexpressed Fhl1p strain exhibited increases in the polysome and monosome levels, showing improved translation activities.

**Conclusion:**

This study illustrated that the transcription factor Fhl1p could effectively enhance recombinant protein expression in *P. pastoris*. Furthermore, we provided the evidence that overexpressed Fhl1p was related to more active translation state.

## Background

The methylotrophic, nonconventional yeast *P. pastoris* is a well-known platform for the production of heterologous proteins intracellularly or extracellularly [1]. Thus far, more than 5000 different proteins have been produced in this yeast due to many advantages, such as high cell density, high yields, and controllable processes [2]. To maximize and optimize the production of recombinant products, recent molecular research has focused on numerous issues, comprising regulating promoter activity [3] and optimizing gene copy number [4] to change the mRNA content of the target gene at the transcriptional level, engineering the folding process and secretory pathway to regulate the posttranslational and transport levels with coexpression of several helper factors. In particular, the helper factors (Ssa1p, Bmh2p, Bfr2p, Pdi1p, Ero1p, Kar2p, etc.) [5–7] are applied to increase the yield of the target protein and to reduce ER stress caused by the overexpression of foreign proteins in the recombinant strains, especially in the multicopy genes expressed strains. Among these helper factors, transcription factors (e.g. Hac1 [8] and Aft1 [9]), which have large potential to regulate the whole protein production process including transcription, translation, posttranslational modification and transportation, are used to boost the antibody fragments and carboxylesterase. In the metabolic flux layer, the glycolytic pathway, tricarboxylic acid (TCA) cycle and nicotinamide adenine dinucleotide (NADH) regeneration pathways are also rationality engineered for expression enhancement [10].

Translation, a process by which a ribosome reads an mRNA template to guide protein synthesis, is critical for gene expression and costs tremendous energy [11, 12]. Improving the mRNA level of target gene is commonly applied to increase the protein expression. However, mRNA and protein levels are imperfectly correlated in yeast and in mammalian cells [11, 13–15]. When optimized promoter and gene dosages are used to enhance the abundance of mRNA, target protein productivity does not linearly increase as expected [16, 17]. Limited by translation efficiency, not all mRNAs are effectively translated into protein and excessive mRNA may be degraded without adequate translation. Nevertheless, few studies have been performed on regulating the translation of yeast strains when engineered to express high protein yields. Therefore, it is meaningful to find a novel transcription factor to regulate translation in engineered strains for industrial production [6].

Herein, we investigated a novel *P. pastoris* transcription factor that was annotated as Fhl1p by sequence homology to *S. cerevisiae* Fhl1p. While little information is currently known about the function of *P. pastoris* Fhl1p, its *S. cerevisiae* homologs have been studied extensively. In *S. cerevisiae*, Fhl1p plays a key role in rRNA processing [18] and ribosomal protein gene expression [19], which have a connection with translation. Defective rRNA processing leads to a severe reduction in the growth rate and a lower rRNA content with the mutation in the *FHL1* gene. Moreover, Fhl1p and two cofactors Ifh1p (a coactivator) and Crf1p (a corepressor) influence the regulation of ribosomal protein (RP) gene transcription via TOR and PKA in yeast [19]. Taken together, the novel transcription factor Fhl1p has the potential to increase the expression of foreign proteins in *P. pastoris*.

In the present study, the enhancing effect provoked by the putative transcription factor Fhl1p was investigated. Overexpression of Fhl1p confered dramatic benefits for the secretion of pectinase and phytase and resulted to higher intracellular mRFP level. Subsequently, gene expression profiles of Fhl1p overexpressed strain (4 pel/AF) and the corresponding control strain with four copy numbers of pectinase gene (4 pel) was further analyzed by RNA-Seq to reveal the regulatory mechanism. Additionally, polysome profiling showed that the efficiency of translation was highly improved when Fhl1p was overexpressed in the pectinase strain. This result may come from the changes of ribosomal small/large subunit biogenesis and translational initiation/elongation, analyzed by RNA-Seq data combined with regulatory sequence analysis tools (RSAT).

## Results and discussion

### *Pichia pastoris* Fhl1p has a conserved Forkhead (FH) domain that binds the DNA sequence

**Fig. 1:**
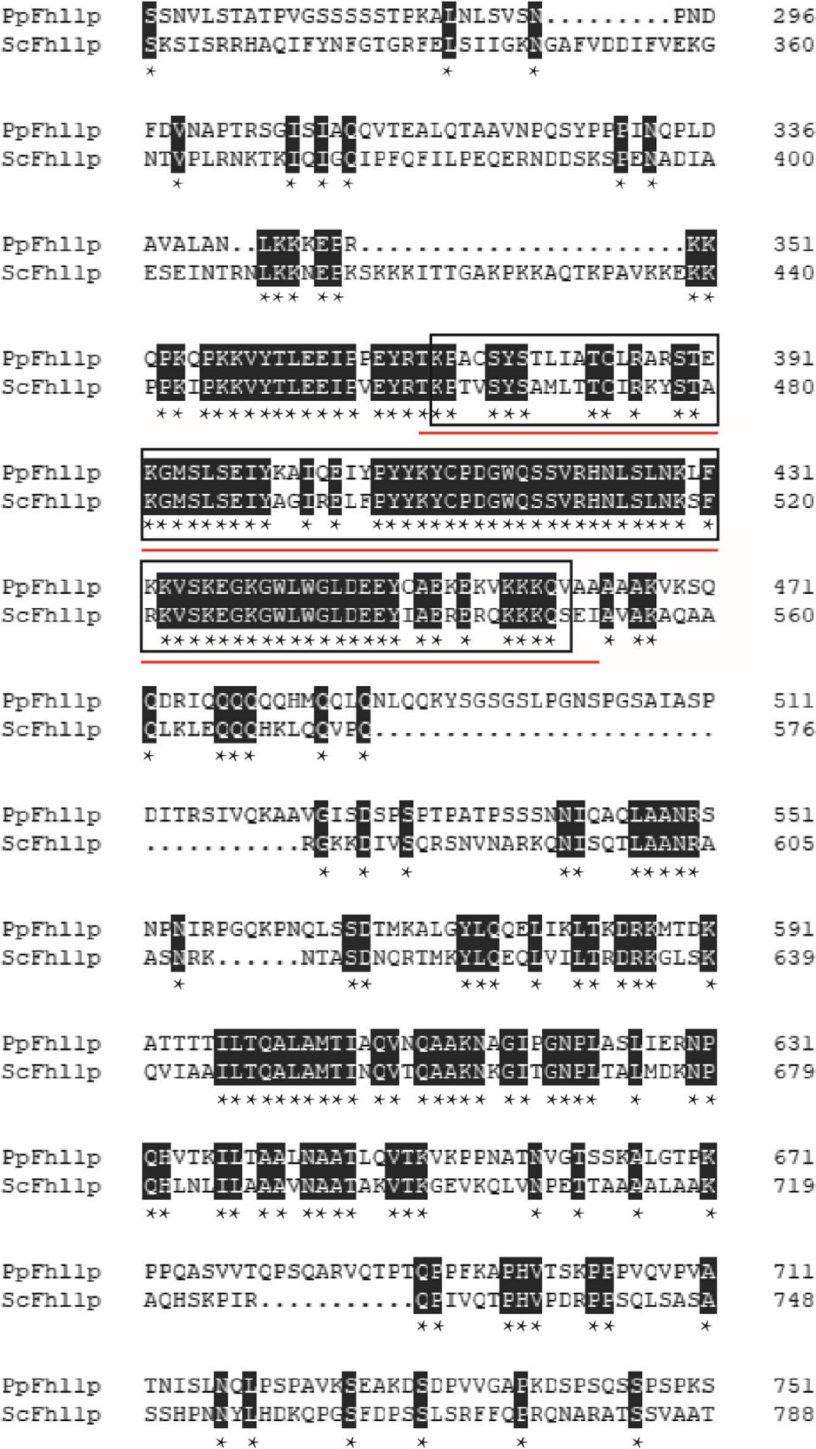
Fhl1p protein sequence comparison. Alignment of amino sequences of *P. pastoris* of Fhl1p and *S. cerevisiae* Fhl1p by the DNAMAN software. The amino acids were shown by one-letter codes. Gaps were introduced to maximize the similarity. In *P. pastoris*, the FH domain between amino acids 372 and 461 and its homologous region were marked by a black frame. A red line below the alignment was used to mark the FH positions of *S*. cerevisiae. An asterisk character was used to indicate positions that have a single, fully conserved residue

The *FHL1* gene of *P. pastoris* (Pp) (GenBank accession number: C4R8K1) codes for a 969-amino-acid protein containing a domain similar to the fork head DNA-binding domain (FH), initially found in the development fork head protein of *Drosophila melanogaster* and in the HNF-3 family of hepatocyte mammalian transcription factors [18]. FH is also known as a “winged helix”, since the structure contains 2 flexible loops or “wings” in the C-terminal region. The PpFhl1p was compared to *S. cerevisiae* (Sc) Fhl1p (GenBank accession number: P39521) using DNAMAN (Lynnon Biosoft Company). The FH of the PpFhl1p between amino acids 372 and 461 were highly homologous to FH of ScFhl1p between amino acids 460 and 552. High conservation was found for the PpFhl1p residues 350–487 (identity 70%) compared to the *S. cerevisiae* ScFhl1p (Fig. 1). The FH domain acts as a DNA bindng site, is known to bind to promoters of yeast ribosomal protein genes in *S. cerevisiae* [20], and likely binds to the same sites in the *P. pastoris* genome. The PpFhl1p also had FHA domain between amino acids 115 and 172, which was a phosphopeptide recognition domain, displaying molecular function on protein binding.

### The effect of *FHL1* overexpression on the enhancement of productivity of recombinant proteins

Alcohol oxidase1 (*AOX1*) promoter which has strong strength when cells are in a medium containing methanol as a sole carbon source was used to overexpress *FHL1* [21]. Phytase, used as an animal feed additive, can remove the phosphate from phytate and reduce feeding costs and pollution caused by fecal excretion of phosphorus. Alkaline pectinases, the pectin depolymerizing enzymes that cleave ɑ-1,4-galacturonosidic linkages of polygalacturonic acid (PGA), have various environment friendly and economic applications in industrial sectors [7, 22]. Increasing the copy number of phytase gene to six and pectinase gene to four enhances the expression of target protein by 141% and 346% relative to original strains and there is a plateau effect when the copy number was increased further, described by Li et al. [7]. Therefore, the phytase and pectinase were selected as model proteins to evaluate the capability of Fhl1p for promoting protein secretion expression. Besides, mRFP is a monomeric red fluorescent protein with excitation 584 nm and emission 610 nm and it is a reporter like EGFP protein for gene expression and protein localization. It was used to assess the enhancing effect of Fhl1p for intracellularly expression protein.

**Fig. 2:**
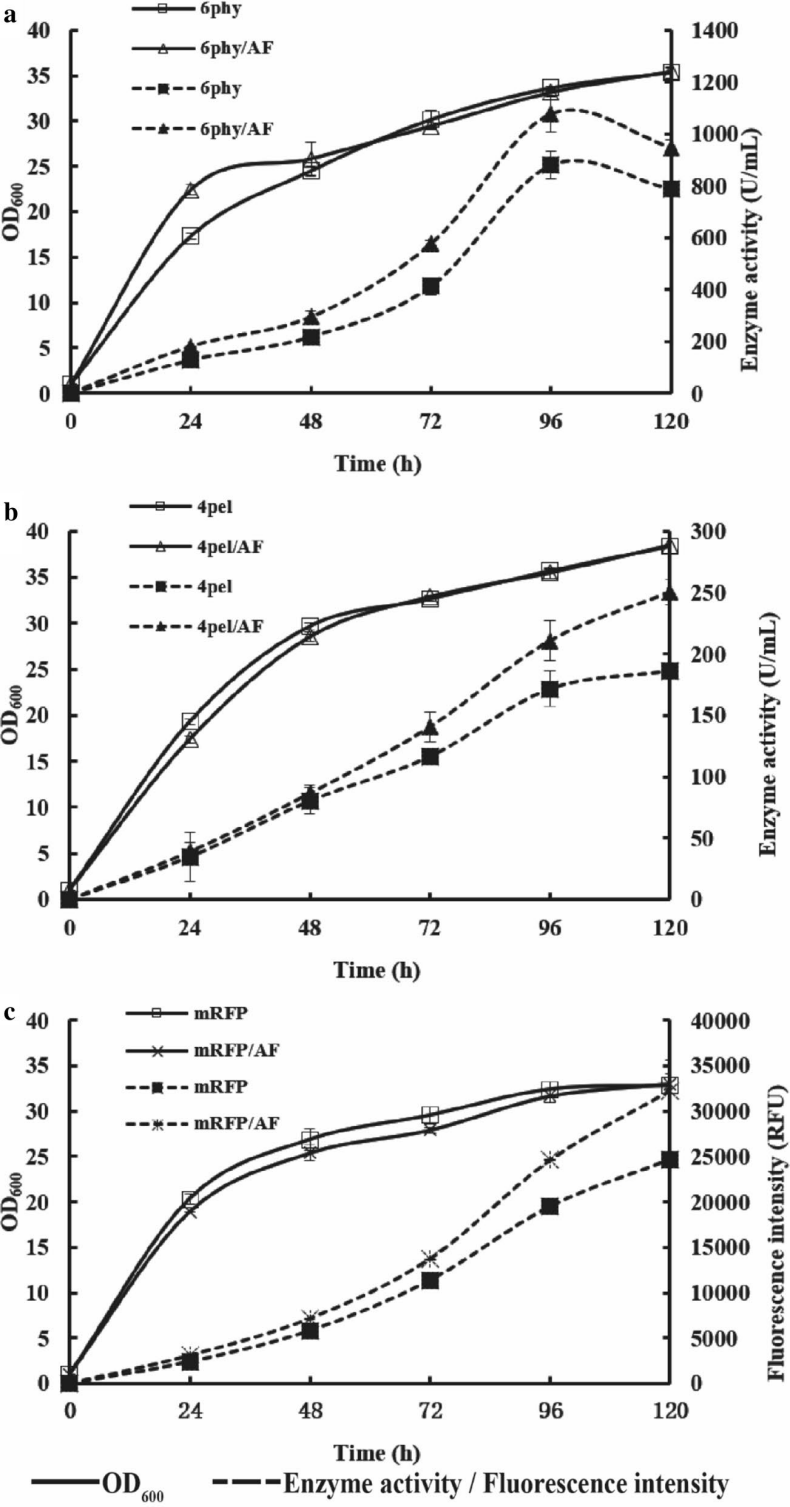
Characterization of the Fhl1p function on promoting the expression of recombinant protein. **a** Phytase expression levels when Fhl1p was overexpressed in the 6 phy strain. **b** Pectinase expression levels when Fhl1p was overexpressed in the 4 pel strain. **c** mRFP expression levels when Fhl1p was overexpressed in mRFP

The strains 6 phy, 4 pel and mRFP harbored six, four, and one copy corresponding gene and were used as the host strain to overexpress *FHL1*, generating 6 phy/AF, 4 pel/AF and mRFP/AF. All strains were cultivated in shake flask and fresh methanol was added to obtain a final concentration of 1% (v/v) every 24 h. For each construct, six clones were tested to take into account clonal variation (Additional file 1: Figure S1), and one representative clone for each target gene were used for further analysis. The integration of *FHL1* into genome was further confirmed through detecting its gene copy number of the recombinant strains. Strain 6 phy, 4 pel and mRFP all had one copy of *FHL1* gene and their overexpressed Fhl1p strains all had two copies (Additional file 1: Figure S2).When 6 phy/AF and 4 pel/AF were compared with the original strain, the phytase and pectinase activity increased by 20% (reaching 947 U/mL, Fig. 2a) and 35% (reaching 250 U/mL, Fig. 2b). In mRFP strain, the overexpression of *FHL1* increased fluorescence by 31% (reaching 32289 RFU, Fig. 2c) compared with that of the original strain. The results suggested that Fhl1p played an important role in the production of heterologous proteins intracellularly or extracellularly. The growth behavior of the *FHL1* overexpressing strain was also considered. Neither significant changes in final optical density (OD) levels (< 15%, 120 h) nor any alterations were observed in the growth curves (Fig. 2) in shake flask. Given the tremendous energy cost associated with protein synthesis, it is not surprising that global protein synthesis for cell growth is generally suppressed under cell burden in the overexpression of a foreign protein [23, 24]. It was an example that yeast strain expressing xylanase had lower growth when HAC1 was overexpressed [25]. The engineered strains 4 pel/AF, mRFP/AF and 6 phy/AF did not show significant declines in growth, may due to modest enhancement with production of target proteins which needed less energy.

There was another positive effect on the protein amount when Fhl1p was overexpressed in 4 pel/AF and mRFP/AF. The protein levels of pectinase in 4 pel and 4 pel/AF were 1.06 ± 0 and 1.44 ± 0.11 g/L, respectively (Additional file 1: Figure S3). Meanwhile, SDS-PAGE was used to compare the protein amount (Additional file 1: Figure S4). The color of mRFP/AF was redder than that of mRFP (Additional file 1: Figure S5). Furthermore, the transcription of *FHL1* and the three target genes were measured (Additional file 1: Figure S6). Not surprisingly, transcription levels of *FHL1* were sharply increased in stains 4 pel/AF, 6 phy/AF and mRFP/AF compared to the corresponding control strain and log2 ratio is 6.66, 2.79 and 7.76, respectively.

### *Pichia pastoris* Fhl1p regulates biological processes

To further investigate the factors enhancing the expression of recombinant proteins, transcription patterns of the 4 pel and 4 pel/AF were analyzed using RNA-Seq. In total, we obtained 48,094,836 reads within the samples cultivated with methanol as the substrate. Of the total reads, 96.52% could be mapped to the *P. pastoris* genome, of which 88.38% were mapped to unique match and 8.14% were mapped to multi-position match. The remaining 3.49% of the total reads were of poor quality and were discarded.

Compared with 4 pel, more genes were upregulated (782 genes) than downregulated (114 genes) in the 4 pel/AF (false discovery rates (FDR) < 0.001 and value of |log2 ratio| ≥ 1 were used to determine the statistical significance of gene expression; Additional file 2). AmiGO GO Slimmer was used to analyze enriched genes; among the upregulated genes, 100 different biological processes including rRNA processing processes (66 hits), transcription from RNA polymerase II promoter processes (52 hits), response to chemical processes (50 hits), ion transport processes (42 hits), transmembrane transport (42 hits), cellular response to DNA damage stimulus processes (40 hits) (Additional file 2), were found to be under the regulation of Fhl1p. Ribosomal small subunit biogenesis processes (31 hits), ribosomal large subunit biogenesis processes (29 hits), translational elongation processes (7 hits), and translational initiation processes (6 hits) were directly related to translation. In addition to translation, a high number of biological processes related to protein folding and secretory pathway machinery [26] were found (Additional file 3), and most of these genes were upregulated. The upregulated genes included 36 for protein targeting, 20 for Golgi vesicle transport, 13 for response to oxidative stress, 12 for folding, 3 for glycosylation, 13 for vesicle organization, 18 for regulation of transport, 10 for exocytosis, etc. In particular, GO Term Finder results showed that Fhl1p had an important influence on rRNA processing processes (Additional file 2), indicating PpFhl1p had the similar function to ScFhl1p [18].

**Fig. 3:**
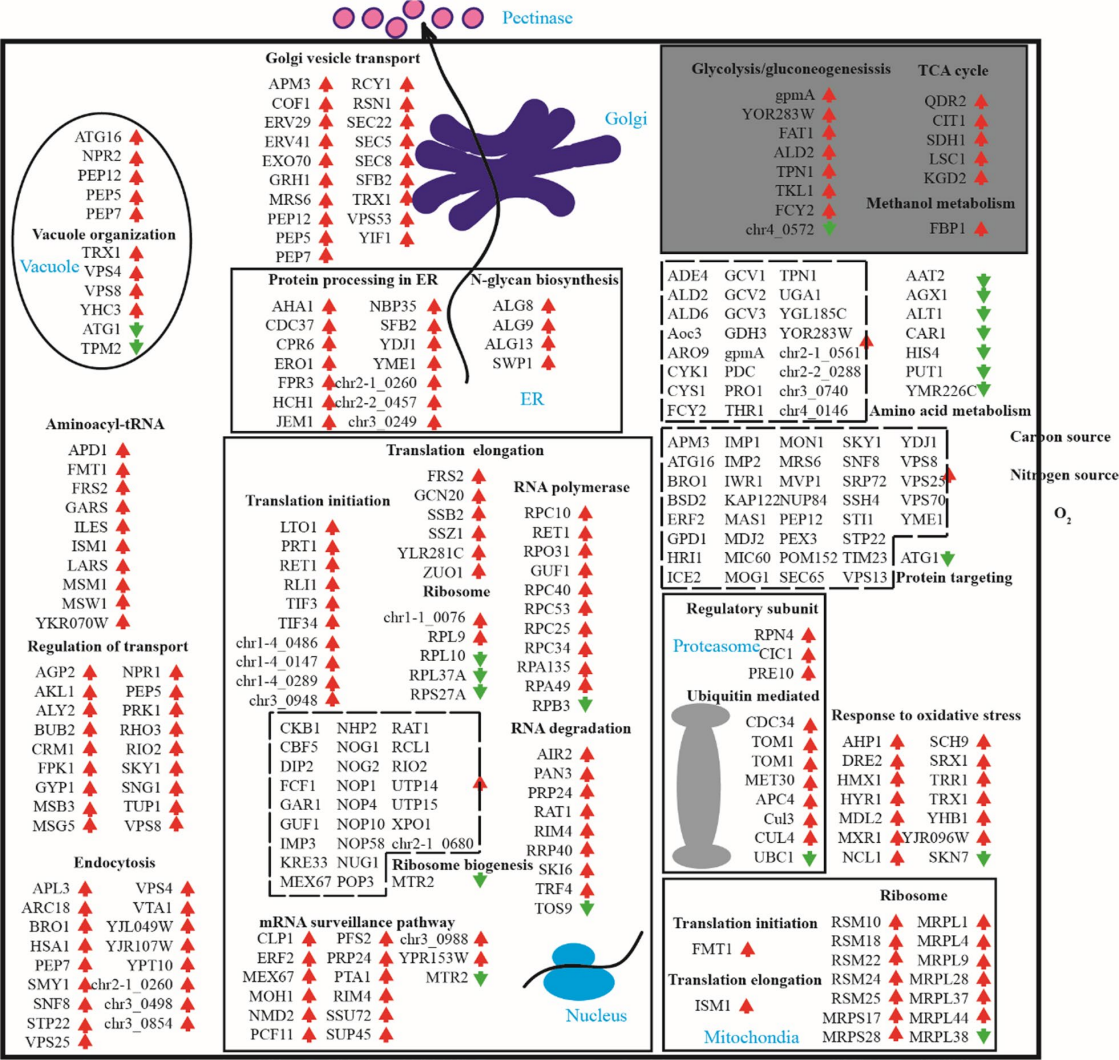
Analysis of differential gene expression of the 4 pel/AF in comparison to 4 pel strain. Red arrows (↑) indicate an increasing and green arrows (↓) indicate a decreasing of the corresponding transcriptional levels in the methanol induction phase

Additionally, we also summarized transcriptional changes from various protein synthesis pathways that were differentially expressed for more detailed discussion (Fig. 3). More information about the annotation of genes was listed in Additional file 4. Translation is extremely important for gene expression. Accordingly, a few genes involved in ribosomal protein, ribosomal biogenesis, translation initiation, etc., were considered. In Fig. 3, the ribosomal protein genes *RPS22* and *RPL9* were upregulated, whereas *RPL10*, *RPL37A* and *RPS27A* were downregulated. Many proteins genes involved in ribosomal biogenesis showed increased levels in the overexpression strain, except for *MTR2*. Translation initiation-related genes, including factor eIF2 gene (*chr1*-*4_0486*), factor eIF3 genes (*TIF34* and *chr1*-*4_0147*), factor eIF-5 gene (*EIF5*), factor 3 subunit B gene (*PRT1*), factor 3 subunit H gene (*chr3_0948*), factor 3 subunit F gene (*chr1*-*4_0289*), *RLI1*, essential iron-sulfur gene required for ribosome biogenesis, and so on were all upregulated. The translation elongation-related genes that were upregulated included the following: *Frs2,* which produces an alpha subunit of cytoplasmic phenylalanyl-tRNA synthetase, *Gcn20*, which produces a positive regulator of the Gcn2p kinase activity, *Ssb2* and *Zuo1*, which produce cytosolic ribosome-associated chaperones, *Ssz1*, which produces a protein that interacts with a protein produced by *Zuo1* (a *DnaJ* homolog) to form a ribosome-associated complex. Yeast protein Ssb2p, which is a homolog of Hsp70, directly interacts with the ribosome in close proximity to the ribosomal tunnel exit [27, 28]. Together with the ribosome-associated complex (RAC), consisting of Zuo1p and Ssz1p, Ssb2p facilitates the folding of newly synthesized polypeptides emerging from the ribosomal tunnel [29–31]. Yeast strains lacking Ssbp or Racp contain reduced levels of assembled ribosomal particles [29, 32–34]. To summarize, these findings indicated that ribosome content could be increased by overexpression of Fhl1p. Interestingly, the mitochondrial ribosomal proteins genes *RSM10,* *RSM18,* *RSM22, RSM24,* *RSM25, MRPS17, MRPS28, MRPL1, MRPL4, MRPL9, MRPL28, MRPL37* and *MRPL44*, but not *MRPL38,* were also upregulated. A translation initiation related gene *Fmt1*, which produces a protein catalyzing the formylation of initiator Met-tRNA in mitochondria, and a translation elongation related gene *Ism1*, which produces a protein as a mitochondrial isoleucyl-tRNA synthetase, were both upregulated, giving more evidence of improved mitochondrial translation. According to report described by Suhm et al. [35], mitochondrial translation impacts cytoplasmic proteostasis and nuclear gene expression. So we speculated that overexpression of Fhl1p produces active mitochondrial translation to relieve ER stress with possible perturbations on expression of recombinant protein.

Translation requires large amounts of energy, so the TCA cycle and glycolysis were the focus for ATP biosynthesis and methanol metabolism, which had a massive impact on the expression of the recombinant protein. Methanol was the sole carbon and energy source, and its utilization was important. However, *CAT1*, *AOX1*, and *FLD1* were slightly downregulated (value of |log2 ratio| < 1), and only *FBP1*, a gene related to fructose-1,6-bisphosphatase which is a key regulatory enzyme in the gluconeogenesis pathway, changed significantly. This indicated that methanol utilization almost did not change. Increased flux towards the glycolytic pathway, the TCA cycle had positive effects on recombinant strains previously discussed [36]. *Cit1*, which produces a citrate synthase, *Sdh1*, which produces a flavoprotein subunit of succinate dehydrogenase (Sdh1p, Sdh2p, Sdh3p, Sdh4p), *Lsc1*, which produces an alpha subunit of succinyl-CoA ligase, *Kgd2*, which produces a component of the mitochondrial alpha-ketoglutarate dehydrogenase, were all up-regulated. Except for enolase produced by gene *chr4_0572*, glycolysis related genes were upregulated. Besides, *GPD1* and *GDH3* which produces proteins acting on the link between metabolism and the reduction of NADP^+^ were also chosen as factors to enhance recombinant protein production. As the synthesis of these amino acids requires reduced NADPH, it was beneficial to enhanced reduction of the NADP/H pool on recombinant protein production [37, 38].

We also focused on the transcription level of UPR-related genes which had a prominent effect on the host cell physiology during recombinant protein production [39–41]. *Hac1* (*PAS_chr1*-*1_0381*), which produces a bZIP transcription factor that regulates the unfolded protein response, *Pdi1* (*PAS_chr4_0844*), which produces a protein disulfide isomerase, *Kar2* (*PAS_chr2*-*1_0140*), which produces an ATPase involved in protein import into the ER and also acts as a chaperone to mediate protein folding, were slightly downregulated (value of |log2 ratio| < 1).

Since PpFhl1p had FH domain and likely binded to the same site of ScFhl1p, we used binding motif GACGC [42] of ScFhl1p as putative PpFhl1p binding site to find gene promoters which contains this motif sequence in *P. pastoris* genome by RSAT. 2245 genes with promoters containing such motif site were available and a total of 1968 genes were annotated [43] and mapped to broader parent terms, Gene Ontology (GO) slim terms, using AmiGO GO Slimmer [44]. Thus, 100 different biological processes comprising transcription from RNA polymerase II promoter processes (161 hits), response to chemical processes (149 hits), cellular response to DNA damage stimulus processes (140 hits), mitotic cell cycle processes (132 hits), and transmembrane transport processes (129 hits) (Additional file 5) were found under the regulation of PpFhl1p. We also found that 86 genes for rRNA processing. Both term enrichment results from RNA-Seq and RSAT showed PpFhl1p was an activator of ribosome biosynthesis processing. Similarly, mitochondrial translation genes (36 hits) were also regulated by PpFhl1p. These data indicated that not only cytoplasmic translation but also mitochondrial translation was enhanced when Fhl1p was overexpressed.

In total, 38% of the upregulated genes and 43% of the downregulated genes contain at least one putative Fhl1p binding site in their promoters (compared to 13% of total *P. pichia* genes), suggesting that both upregulation and downregulation were direct consequences of *FHL1* overexpression. To validate quantitative results from the RNA-Seq analysis, 4 genes were selected for quantitative RT-PCR analysis, and information about the primers, plasmids and strains were included in Additional file 6.

### The translation activity was promoted by *FHL1* overexpression

**Fig. 4:**
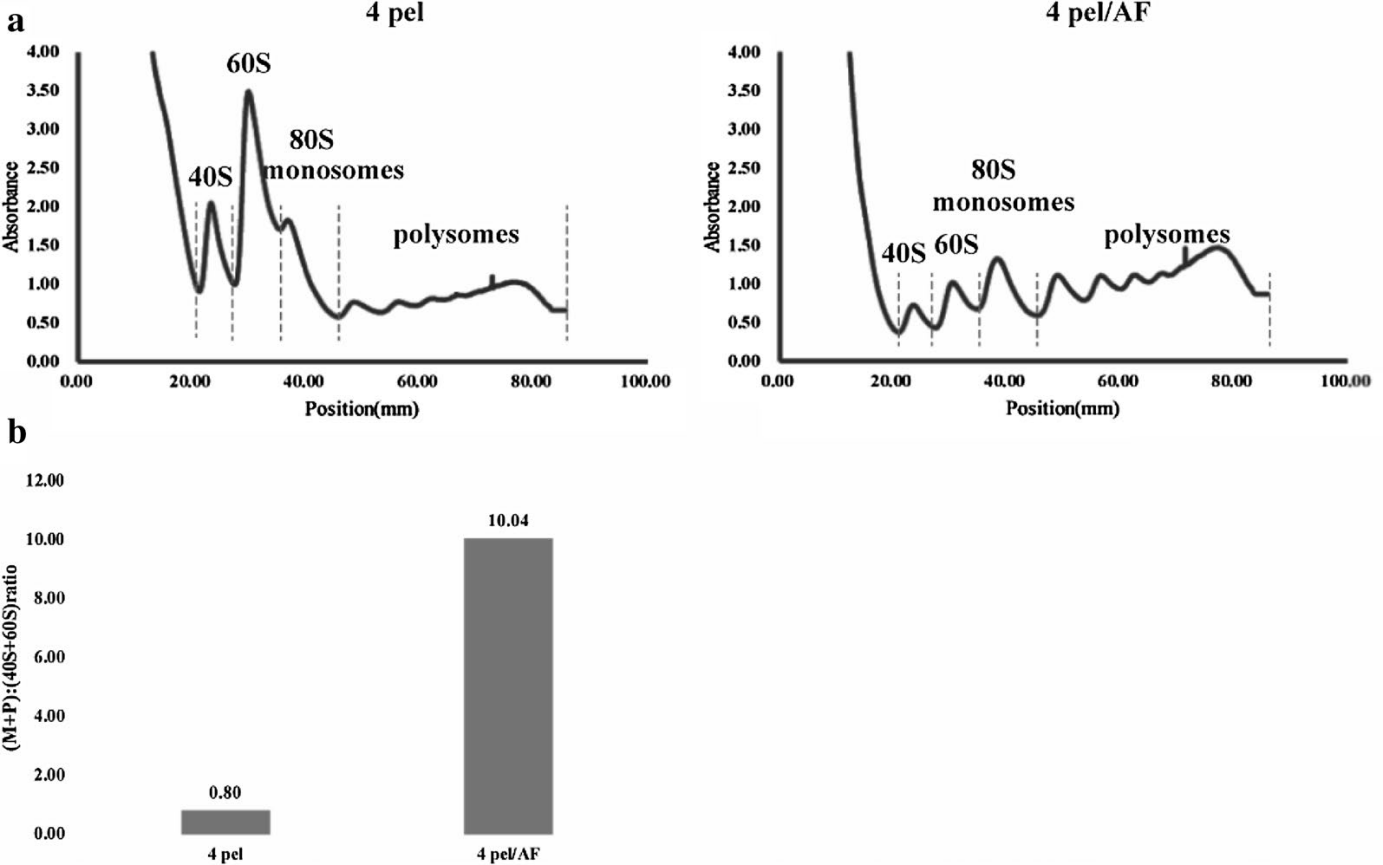
Polysome profiles and (M + P):(40S + 60S) ratios for strains grown in methanol conditions. **a** Representative polysome profiles and **b** a chart presenting (M + P):(40S + 60S) ratios of the different strains. Corresponding peaks (40S, 60S, 80S/monosomes and polysomes) are indicated in the polysome profile. (M + P):(40S + 60S) ratios were calculated from areas beneath the profile curve using ImageJ

Polysome profiling experiments were performed using 4 pel and 4 pel/AF and the translational status of a cell was characterized according to the distribution of ribosomes across the mRNA pool [45]. Individual subunits (40S and 60S), which are defined as the ribosomal small subunit and the ribosomal large subunit, monosomes (80 S) or polysomes (two or more ribosomes that are associated with a given mRNA transcript) are shown by profile curves indicating the proportion of ribosomes showing different translation conditions in the two yeast strains (Fig. 4a). mRNAs that are associated with polysomes are more highly-translated than mRNAs associated with monosomes [46], reflecting a relative measure of translational activity at a cellular level. The P:M ratio which refers to the ratio of polysome to monosome peak areas was traditionally therefore established as a relative measure of translational activity at the cellular level [47, 48]. But cells can be in an active translational state even though mRNAs were associated with monosomes [49, 50]. Thus, the ratio of polysomes and monosomes to 40S and 60S ((M+P):(40S+60S)) was chosen to represent the translational state. The 4 pel/AF yeast strain exhibited an increasement in the polysome and monosomes peak areas with a corresponding decrease in the 40S and 60S peak areas (Fig. 4), indicating a more active translation in this strain than in 4 pel strain. The (M+P):(40S+60S) ratio of 4 pel/AF was 10.04 which was significantly higher than the ratio of 4 pel, which was 0.80 (Fig. 4b). This result and the normal growth of the overexpression strain indicated that the transcription factor Fhl1p improved the content of the factors related to translation and was certified by RNA-Seq. In accordance with the above mentioned data, Fhl1p was found in the upstream region of several genes relevant to rRNA processing and ribosomal subunit formation, conferring the ability to induce translation activity and supporting the hypothesis of a role of Fhl1p in translation. According to investigating the transcription levels of *pel*, *phy* and *mRFP* genes, there were no significant changes of target genes in original strain and overexpressed Fhl1p strains (Additional file 1: Figure S7). Consequently, Fhl1p had a positive function at translation, not transcription layer.

## Conclusions

The production enhancing effect of Fhl1p was confirmed in the overexpressing strains, produced 20–35% higher pectinase, phytase and mRFP. Fhl1p overexpression performance has been validated at 15L high cell density fed batch cultures in a parallel study (data not shown). As a novel factor, the ortholog of the ScFhl1p, Fhl1p had a positive impact not only on the intracellular expression but also on the extracellular expression of recombinant proteins. A genome-wide analysis of putative Fhl1p binding sites was carried out to demonstrate the function of this transcription factor, finding that Fhl1p involved in protein processing in the ER, glycolysis/gluconeogenesis and TCA cycle, in addition to ribosome biosynthesis. These findings were supported by prediction of RSAT, which had results similar to RNA-Seq data. Polysome profiling experiments further proved that overexpression of Fhl1p was useful to promote the translation efficiency. More interestingly, translation not only in the cytoplasm but also in the mitochondria was enhanced.

## Materials and methods

### Strains, plasmids and media

*Escherichia coli* TOP10 (Invitrogen, Carlsbad, CA, USA) was used for DNA manipulation, gene cloning and sequencing. *P. pastoris* strain GS115 (his4) was used to construct the expression strain. GS115 and recombinant *P. pastoris* strains were cultivated in either YPD medium (1% yeast extract, 2% dextrose, and 2% tryptone) or BMGY/BMMY medium (1% yeast extract, 2% dextrose, 1.34% YNB, 4 × 10^−5^% biotin, 100 mM potassium buffer, and 1% glycerol or 0.5% methanol).

The *FHL1* gene [Gene ID: 8201375] was obtained from genomic DNA of the *P. pastoris* strain GS115 using the appropriate primer pair, *FHL1*-F and *FHL1*-R. PCR products were ligated into the pPIC6αC plasmid. Both plasmids possess the *AOX1* promoter sequence and results in the vectors pPIC6C-*FHL1* and pPICZA-*FHL1*. To generate the mRFP expression plasmid pZHKA-mRFP, the mRFP fragment was amplified by PCR from pFA6a-mRFP-ura4MX6 [51] using the fusion primer pair, mRFP-F and mRFP-R, and was assembled with the other fragment generated from pZHKA.

Strains, vectors and primers used in the present study are summarized in Additional file 6.

### Yeast transformation and regeneration of selectable markers

The plasmid pmRFP was linearized with *Kpn*2I (Thermo Scientific, Waltham, MA) and transformed into GS115 competent cells, creating GS115/mRFP (mRFP). It was then transferred by pPIC6C-*FHL1* and resulted in GS115/mRFP/*AOX*-*FHL1* (mRFP/AF). The transformation method used was the electroporation method described by Cregg [52] and the following parameters were used: 1.5–2.0 kV, 25 μF and 200 Ω. The vector pPICZA-*FHL1* was transferred to the phytase strain GS116/P-6C (6phy) [53] and the pectinase strain GS115/Pel-4C, which were used to generate the 6 phy/AF and 4 pel/AF strains. The transformed cells were selected on YPDZ or YPDB plates. The integration of these plasmids into the yeast genome was verified by PCR using matched primer pairs.

### Shaking flask culture

A single colony of each recombinant *P. pastoris* strain was transformed into 25 mL BMGY medium and incubated for approximately 24 h at 30 °C until the OD_600_ of the culture reached 2–6. The cells were then harvested by centrifugation (10,000×*g*, 10 min, 4 °C) and resuspended in 100 mL BMMY medium in 250-mL baffled flasks to an OD_600_ = 1. Methanol was added into the medium every 24 h to a final concentration of 1.0% to maintain induction. Meanwhile, samples were taken every 24 h to determine the pectinase activity. All strains that expressed the pectinase gene, phytase gene, mRFP gene and *FHL1* under pAOX1 were cultivated in BMMY medium containing 1% methanol in three biological replicates.

### Fluorescence measurements

The fluorescence of mRFP was determined using 200 µL of the cell mixture with 0.1 M phosphate buffered saline (PBS;Life Technology). Measurements were performed in a microplate on a SynergyMX plate reader (Biotek), applying the following settings: excitation 584 nm and emission 610 nm [54].

### Assay of phytase and pectinase activity

Phytase activity was determined as described previously [7]. The enzyme reaction mixture was preheated at 37 °C for 5 min. Then, 5.0 mM sodium phytate (pH 5.5) was added, and the mixture was incubated at 37 °C for 30 min. The reaction was stopped by adding a coloration solution. The absorbance of the mixture was quantified at 415 nm.

Pectinase activity was routinely determined by measuring the absorbance change at 235 nm with 2 mg/mL of polygalacturonic acid (PGA; Sigma) as the substrate in 50 mM glycine–NaOH (pH 10.0) buffer containing 1 mM CaCl_2_. One unit (U) of pectin lyase activity was defined as the amount of enzyme that is required to produce unsaturated oligogalacturonide equivalent to 1 μmol of unsaturated galacturonic acid per minute at a molecular extinction coefficient of 4600 M^−1^ cm^−1^ at 235 nm [55].

### Regulatory sequence analysis tools (RSAT)

A series of computer programs are developed for the analysis of regulatory sequences, with a special focus on yeast. These tools are publicly available on the web (http://pedagogix-tagc.univ-mrs.fr/rsat/). Basically, three classical problems can be addressed: (a) search for known regulatory patterns in the upstream regions of known genes; (b) discovery of unknown regulatory patterns within a set of upstream regions known to be co-regulated; (c) search for unknown genes potentially regulated by a known transcription factor. Each of these tasks can be performed on basis of a simple (string) or more refined (matrix) description of the regulatory patterns. A feature-map program automatically generates visual representations of the positions at which patterns were found. The site also provides a series of general utilities, such as generation of random sequence, automatic drawing of XY graphs, interconversions between sequence formats, etc. Several tools are linked together to allow their sequential utilization (piping), but each one can also be used independently by filling the web form with external data. This widens the scope of the site to the analysis of nonregulatory and/or non-yeast sequences [56]. We used known regulatory patterns of ScFhl1p GACGC to search for unknown genes potentially regulated by a known transcription factor. RSAT genome-scale DNA-pattern in strings of subcategory pattern matching was used to search for binding sites within − 1000 bp upstream of the *P. pichia* GS115 coding sequences. The default settings were applied.

### AmiGO GO Slimmer

AmiGO is a web application that allows users to query, browse and visualize ontologies and related gene product annotation (association) data. AmiGO can be used online at the Gene Ontology (GO) website to access the data provided by the GO Consortium; it can also be downloaded and installed to browse local ontologies and annotations. AmiGO is free open source software developed and maintained by the GO Consortium. Its functionality contained blast, term enrichment and GO Slimmer, etc. The function of the GO Slimmer tool is to remap granular, specific annotations up to a user-specified set of high-level terms. This subset of terms, referred to as a GO slim, provides a useful overview of a dataset and facilitates the reporting and analysis of large result sets, such as GO annotations to a genome or microarray expression data [57]. In this study, AmiGO GO Slimmer was utilized to map genes into GO slim terms according to their biological process. SGD was used as a database filter (Evidence Code: all), and Yeast GO slim was used as a preexisting GO slim set. The advanced results option was used to display gene products and counts for each slim term.

### Polyribosome profile analysis

For polyribosome preparation, 50 mL cultures of 4 pel/AF and 4 pel were grown to a log phase (OD600 ~ 0.8–1.0), cycloheximide (CHX) was added to a final concentration of 100 μg/mL, and then cultivated for 15 min. The cells were chilled immediately on ice. After centrifugation at 3000×*g* for 3 min at 4 °C, the cell pellets were washed once with 5 mL ice-cold lysis buffer (140 mM KCl, 5 mM MgCl2·6H_2_O, 20 mM Tris, pH 7.4, 1% Triton X-100, 1 mM DTT, 100 μg/mL CHX, and 1 mM PMSF) and resuspended in 1 mL cold lysis buffer, which was transferred to a 2 mL centrifuge tube. Then, 750 μL of RNase-free acid-washed glass beads were added, and the cells were vortexed for 0.5 min in a BeadBeater for 6 cycles. Lysates were spun down briefly to reduce the foam. Whole lysates were transferred to a 1.5 mL centrifuge tube. After centrifugation in a microcentrifuge for 5 min at 10,000×*g* and 4 °C, the absorbance at 260 nm was measured, and samples (20 units at OD 260 nm) were loaded onto a sucrose gradient (high-salt 10–45% sucrose gradient buffer containing 50 mM Tris–HCl, pH 7.4, 800 mM KCl, 15 mM MgCl_2_·6H2O, and 100 μg/mL cycloheximide). The gradients were centrifuged in a Beckman SW40 rotor at 39,000 rpm for 2 h, and gradient fractions were read on an ISCO UA-5 absorbance detector at an absorbance of 260 nm [58].

### RNA-seq analysis and RT-PCR

Total RNA was extracted from strains 4 pel and 4 pel/AF grown in BMMY for 120 h. Approximately 1 × 10^7^ cells were used for the total RNA extraction using the hot acidic phenol method [59]. RNA concentrations were quantified by measuring the absorbance at 260 nm using a NanoDrop 2000c (Thermo Scientific, Waltham, MA, USA). Total RNA was isolated and reverse transcribed into cDNA to generate an indexed Illumina library, followed by sequencing at the Beijing Genomics Institute (Beijing, China) using a BGISEQ-500 platform. High-quality reads were aligned to *P. pichia* reference genome and gene by HISAT and Bowtie2. The gene expression levels were normalized to the FPKM (fragments per kilobase million) reads by RNA-seq by an expectation maximization algorithm. Significant differential expression of a gene was defined as a ≥two fold expression difference vs. the control with FDR < 0.001. DEGs were analyzed by Gene Ontology. The enrichment degrees of DEGs were analyzed using Kyoto Encyclopedia of Genes and Genomes annotations. The qPCR and real-time PCR (RT-PCR) assays were repeated three times per sample. RT-PCR data were normalized using the *GAPDH* gene (i.e., housekeeping gene) as an endogenous control. Changes in expression levels of genes selected based on their regulation patterns were confirmed using quantitative real-time PCR.

### Statistical analysis

All data generated or analysed during this study are included in this article (and its additional files). Differences between groups were tested for statistical significance by using a two tailed by unpaired T-test in Microsoft Excel 2010 (Microsoft, Redmond, Washington). Differences were considered significant at P < 0.05.

## Supplementary information

**Additional file 1: Figure S1.** The enzyme activity and RFU of pectinase strain (A), phytase (B) and mRFP (C) strain with overexpression of Fhl1p after 120 h of induction with methanol. Six constructed clones were tested to assess clonal variation. Box-plot is used to show the distribution of expression level. Medians are shownd by horizontal bars. Statistical significance was examined using a two tailed by unpaired T-test analysis. *P < 0.05, **P < 0.01, ***P < 0.001. **Figure S2.** The copy number of *FHL1* gene and *mRFP* gene. **Figure S3.** Effect of overexpression in protein content of pectinase and phytase after 120 h of induction with methanol. Statistical significance was examined using a two tailed by unpaired T-test analysis. *P < 0.05, **P < 0.01, ***P < 0.001, ns: no significant difference. **Figure S4.** SDS-PAGE of pectinase and phytase after 120 h of induction with methanol. **Figure S5.** The comparison of mRFP and mRFP/AF in color. **Figure S6.** The transcription levels of *FHL1* in strains harboring pectinase, phytase and mRFP after 120 h of induction with methanol. Statistical significance was examined using a two tailed by unpaired T-test analysis. *P < 0.05 and |log2ratio| ≥ 1, **P < 0.01 and |log2ratio| ≥ 1, ***P < 0.001 and |log2ratio| ≥ 1. **Figure S7.** Effect of overexpression Fhl1p on transcription levels of pectinase, phytase and mRFP after 120 h of induction with methanol. Statistical significance was examined using a two tailed by unpaired T-test analysis. *P < 0.05 and |log2ratio| ≥ 1, **P < 0.01 and |log2ratio| ≥ 1 , ***P < 0.001 and |log2ratio| ≥ 1, ns: no significant difference.

**Additional file 2.** List of significantly upregulated and downregulated genes in 4 pel/AF in comparison to 4 pel. GO term enrichment was analyzed using AmiGO GO Slimmer [44]. Significantly upregulated and downregulated GO terms were determined by GO term Finder (P < 0.01).

**Additional file 3.** The changed biological processes of the overexpression strain 4 pel/AF in comparison to 4 pel.

**Additional file 4.** The annotation of proteins from various protein synthesis pathways that are differentially expressed.

**Additional file 5.** *P. pastoris* genes with putative Fhl1p binding site(s). Search field: − 1000 bp upstream of the *P. pastoris* GS115 coding sequences. The binding motif of ScFhl1p is GACGC. Genes were categorized into biological function GO-Terms using AmiGO GO Slimmer (Yeast GO slim set, [44]).

**Additional file 6.** Primers used for the construction of engineered strains and the verification of RNA-Seq data by RT-PCR.

## Data Availability

The dataset supporting the conclusions of this article is included within the article.
