# Supplementary material for: Fhl1p protein, a positive transcription factor in Pichia pastoris, enhances the expression of recombinant proteins
Source: Microb Cell Fact. 2019 Nov 29;18:207. doi: 10.1186/s12934-019-1256-0 (PMC6884909; doi:10.1186/s12934-019-1256-0)
Supplement: Supplementary file 1 — Additional file 1: Figure S1. The enzyme activity and RFU of pectinase strain (A), phytase (B) and mRFP (C) strain with overexpression of Fhl1p after 120 h of induction with methanol. Six constructed clones were tested to assess clonal variation. Box-plot is used to show the distribution of expression level. Medians are shownd by horizontal bars. Statistical significance was examined using a two tailed by unpaired T-test analysis. *P < 0.05, **P < 0.01, ***P < 0.001. Figure S2. The copy number of FHL1 gene and mRFP gene. Figure S3. Effect of overexpression in protein content of pectinase and phytase after 120 h of induction with methanol. Statistical significance was examined using a two tailed by unpaired T-test analysis. *P < 0.05, **P < 0.01, ***P < 0.001, ns: no significant difference. Figure S4. SDS-PAGE of pectinase and phytase after 120 h of induction with methanol. Figure S5. The comparison of mRFP and mRFP/AF in color. Figure S6. The transcription levels of FHL1 in strains harboring pectinase, phytase and mRFP after 120 h of induction with methanol. Statistical significance was examined using a two tailed by unpaired T-test analysis. *P < 0.05 and |log2ratio| ≥ 1, **P < 0.01 and |log2ratio| ≥ 1, ***P < 0.001 and |log2ratio| ≥ 1. Figure S7. Effect of overexpression Fhl1p on transcription levels of pectinase, phytase and mRFP after 120 h of induction with methanol. Statistical significance was examined using a two tailed by unpaired T-test analysis. *P < 0.05 and |log2ratio| ≥ 1, **P < 0.01 and |log2ratio| ≥ 1 , ***P < 0.001 and |log2ratio| ≥ 1, ns: no significant difference. [file 12934_2019_1256_MOESM1_ESM.docx]

A

C

B

**Additional file 1:****Figure S1 The enzyme activity and RFU of pectinase strain (A), phytase (B) and mRFP (C) strain with overexpression of Fhl1p after 120 h of induction with methanol**. Six constructed clones were tested to assess clonal variation. Box-plot is used to show the distribution of expression level. Medians are shownd by horizontal bars. Statistical significance was examined using a two tailed by unpaired T-test analysis. *P < 0.05, **P < 0.01, ***P < 0.001

**Figure S2** **The copy number of *FHL1* gene and *mRFP* gene**

**Figure S3** **Effect of overexpression in protein content of pectinase and phytase after 120 h of induction with methanol.** Statistical significance was examined using a two tailed by unpaired T-test analysis. *P < 0.05, **P < 0.01, ***P < 0.001, ns: no significant difference


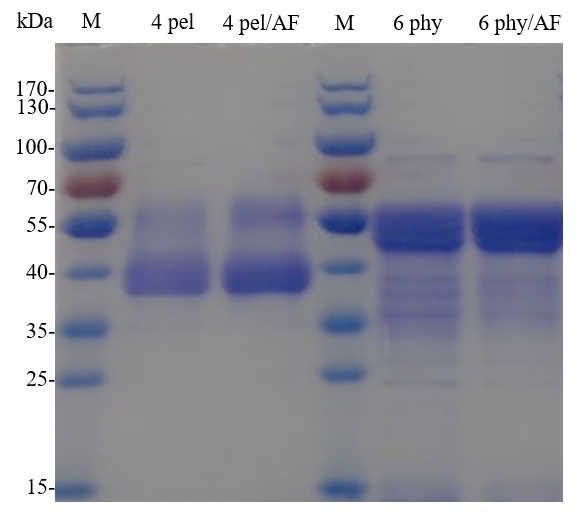


**Figure S4** **SDS-PAGE of pectinase and phytase after 120 h of induction with methanol**


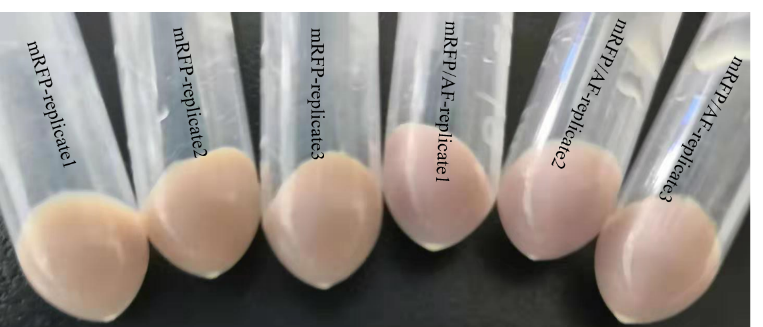


**Figure S5 The comparison of mRFP and mRFP/AF in color**

**Figure S6 The transcription levels of *FHL1* in pectinase, phytase and mRFP expression strains after 120 h of induction with methanol.** Statistical significance was examined using a two tailed by unpaired T-test analysis. *P<0.05 and |log2ratio|≥1,**P<0.01 and |log2ratio|≥1, ***P<0.001 and |log2ratio|≥1

**Figure S7 Effect of overexpression Fhl1p on transcription levels of pectinase, phytase and mRFP after 120 h of induction with methanol.** Statistical significance was examined using a two tailed by unpaired T-test analysis. *P<0.05 and |log2ratio|≥1,**P<0.01 and |log2ratio|≥1, ***P<0.001 and |log2ratio|≥1, ns: no significant difference
