# Supplementary material for: Fhl1p protein, a positive transcription factor in Pichia pastoris, enhances the expression of recombinant proteins
Source: Microb Cell Fact. 2019 Nov 29;18:207. doi: 10.1186/s12934-019-1256-0 (PMC6884909; doi:10.1186/s12934-019-1256-0)
Supplement: Supplementary file 3 — Additional file 3. The changed biological processes of the overexpression strain 4 pel/AF in comparison to 4 pel. [file 12934_2019_1256_MOESM3_ESM.docx]

**Additional File 3: The changed biological processes of the overexpression strain 4 pel/AF in comparison to 4 pel.**

**Table S1** ***P. pastoris* differentially expressed genes related to the translation, folding and secretory pathway in the 4 pel/AF vs. 4 pel strain.**

| Up-regulated genes (4 pel/AF vs. 4 pel) |  |
| --- | --- |
| GO slim term-Biological process | number (genes) |
| GO:0006364 rRNA processing | 66 ( BFR2 CBF5 DBP3 DBP7 DBP8 DBP9 DIM1 DIP2 DRS1 ECM16  FAF1 FCF1 GAR1 HIT1 IMP3 KRE33 KRI1 KRR1 MAK11 MAK16  MRD1 MRH4 NHP2 NOC4 NOG1 NOP1NOP10 NOP14 NOP16  NOP19 NOP2 NOP4 NOP58 NOP7 NSA2 NSR1 NUG1 POP3 PWP1  PXR1 RAT1 RCL1 RIO2 RIX1 RLI1 RPF1 RRB1 RRP1 RRP12 RRP36 RRP40 RRS1SAS10 SKI6 SOF1 SPB1 SSB2 SSZ1 TOM1 TSR3 URB1 UTP11 UTP14 UTP15 UTP30 ZUO1 ) |
| GO:0042274 ribosomal small subunit biogenesis | 31 ( BFR2 DBP8 DIM1 DIP2 ECM16 FAF1 FCF1 IMP3 KRE33 KRI1  KRR1 MRD1 NOC4 NOP14 NOP19 NOP58 NOP7 NSR1 RCL1 RIO2 RRP12 RRP36 RRS1 SAS10 SOF1TOM1 TSR3 UTP11 UTP14 UTP15 UTP30 ) |
| GO:0042273 ribosomal large subunit biogenesis | 29 ( DBP3 DBP7 DBP9 DRS1 HIT1 LTO1 MAK11 MAK16 MRH4  NHP2 NOC2 NOG1 NOP16 NOP2 NOP4 NOP7 NSA2 NUG1 RAT1  RIX1 RLI1 RPF1 RRS1 SDA1 SPB1 SQT1TOM1 URB1 UTP30 ) |
| GO:0006414 translational elongation | 7 ( FRS2 GCN20 ISM1 SSB2 SSZ1 YLR281C ZUO1 ) |
| GO:0006413 translational initiation | 6 ( FMT1 LTO1 PRT1 RLI1 TIF3 TIF34 ) |
| GO:0048193 Golgi vesicle transport | 20 ( APM3 COF1 ERV29 ERV41 EXO70 GRH1 MRS6 PEP12 PEP5  PEP7 RCY1 RSN1 SEC22 SEC5 SEC8 SFB2 TRX1 USO1 VPS53 YIF1 ) |
| GO:0006979 response to oxidative stress | 13 ( AHP1 DRE2 HMX1 HYR1 MDL2 MXR1 NCL1 SCH9 SRX1  TRR1 TRX1 YHB1 YJR096W ) |
| GO:0006457 protein folding | 12 ( AHA1 CDC37 CPR6 ERO1 FPR3 HCH1 JEM1 SSB2 STI1 YDJ1  YME1 ZUO1 ) |
| GO:0006486 protein glycosylation | 3 ( ALG8 ALG9 SWP1 ) |
| GO:0007033 vacuole organization | 9 ( ATG16 NPR2 PEP12 PEP5 PEP7 TRX1 VPS4 VPS8 YHC3 ) |
| GO:0051049 regulation of transport | 18 ( AGP2 AKL1 ALY2 BUB2 CRM1 FPK1 GYP1 MSB3 MSG5 NPR1 PEP5 PRK1 RHO3 RIO2 SKY1 SNG1 TUP1 VPS8 ) |
| GO:0006887 exocytosis | 10 ( EXO70 MSB3 PEP5 PEP7 RCY1 RHO3 SEC2 SEC5 SEC8 SMY1 ) |

**Table S1** ***P. pastoris* differentially expressed genes related to the translation, folding and secretory pathway in the 4 pel/AF vs. 4 pel strain**

| Down-regulated genes (4 pel/AF vs. 4 pel) |  |
| --- | --- |
| GO slim term-Biological process | number (genes) |
| GO:0006364 rRNA processing | 3 ( MRM2 RPL37A RPS27A ) |
| GO:0042274 ribosomal small subunit biogenesis | 1 ( RPS27A ) |
| GO:0042273 ribosomal large subunit biogenesis | 2 ( RPL10 RPL37A ) |
| GO:0006605 protein targeting | 1 ( ATG1 ) |
| GO:0006979 response to oxidative stress | 1 ( SKN7 ) |
| GO:0007033 vacuole organization | 2 ( ATG1 TPM2 ) |
| GO:0006887 exocytosis | 1 ( TPM2 ) |
| GO:0006364 rRNA processing | 3 ( MRM2 RPL37A RPS27A ) |
