# Supplementary material for: Fhl1p protein, a positive transcription factor in Pichia pastoris, enhances the expression of recombinant proteins
Source: Microb Cell Fact. 2019 Nov 29;18:207. doi: 10.1186/s12934-019-1256-0 (PMC6884909; doi:10.1186/s12934-019-1256-0)
Supplement: Supplementary file 6 — Additional file 6. Primers used for the construction of engineered strains and the verification of RNA-Seq data by RT-PCR. [file 12934_2019_1256_MOESM6_ESM.docx]

**Additional File 6 :** The expression changes of some genes observed in the RNA-Seq was confirmed using quantitative real time PCR.

Determination of transcript levels by quantitative real-time PCR was performed using the primers given in Additional Table S2 (Started with RT). *GAPDH* was used as reference gene for normalization. Each transcript was correlated to the *GAPDH* transcript level from the same sample as an internal control. One biological replicate of the 4 pel was used as the reference strain for each relative transcript level determination using the ΔΔCt method. As can be seen in Additional Figure S8, there is a high degree of correlation between RNA-Seq and RT-PCR results.

**Table S2**. Primers, vectors and strains used in this study.

|  |  | **Primer sequences (5’-3’) or a short description of the plasmids** | Endonucleases |
| --- | --- | --- | --- |
| FHL1-F |  | CGCTTCGAAATGATGAGTGTTAGCTCT | *Bst*BI |
| FHL1-R |  | CAAGCGGCCGCCTATTGTTCTGATTTGGCCA | *Not*I |
| mRFP-F |  | CTCTAGTCAAGACTTACAATTAAAATGGCCTCCTCCGAGGACGTC |  |
| mRFP-R |  | GGAACAGTCATGTCTAAGGGGCGCCGGTGGAGTGGCGGC |  |
| ZA-F |  | CCTTAGACATGACTGTTCCTC |  |
| ZA-R |  | TTTAATTGTAAGTCTTGACTAGAGCACGTGAATTCCTCGTTTCGAA |  |
| GAPDH-F |  | TCCAAGGATGCTCCAATGTTCG |  |
| GAPDH-R |  | GGAGTGGACGGTGGTCATCAAA |  |
| RT-HAC-F |  | GTAAAAGAGCAAAGACGGAAGA |  |
| RT-HAC-R |  | TGTTAGTGGCTTTGGCTGATTC |  |
| RT-PDI-F |  | TATTGATGGATTTCCGACGATG |  |
| RT-PDI-R |  | GAACTAACGAAGGCTCATTTAC |  |
| RT-AOX1-F |  | TGGAGACCTCTAATGCCTACGG |  |
| RT-AOX1-R |  | TCTCAATGGCCTTGTCATCCTC |  |
| RT-Hxt2p-F |  | CTTCGCTTCTTCAGCCCTCTAC |  |
| RT-Hxt2p-R |  | GCATTGGGTCGTTATTGTTCAT |  |
| RT-FHL1-F |  | CAACTGGCAGCCAACAGAAGTA |  |
| RT-FHL1-R |  | GCCTGGGTTAGAATCGTCGTAG |  |
| RT-mRFP-F |  | GAGATCAAGATGAGGCTGAA |  |
| RT-mRFP-R |  | ATGGTGTAGTCCTCGTTGTG |  |
| RT-phy-F |  | TGGTTGGGGTAGAATCAC |  |
| RT-phy-R |  | TGCTTCTGAGGAGGATGA |  |
| RT-pel-F |  | ACTACAGGTGGAGAGGGTGGAC |  |
| RT-pel-R |  | GACGATTGAAACATTGGAGACG |  |
| **Plasmids** |  |  |  |
| pHKA |  | To offer the HIS4 gene fragment |  |
| pZHKA |  | To offer the HIS4 gene fragment and zeocin  expression cassette |  |
| pZAF |  | To overexpress FHL1 under AOX1 promoter with zeocin marker |  |
| pPIC6αC |  | Invitrogen |  |
| pBAF |  | To overexpress FHL1 under AOX1 promoter with blasticidin marker |  |
| pPICHKA-(pel)4 |  | To offer the pectinase expression cassette |  |
| pZHKA-mRFP |  | To offer the mRFP expression cassette |  |
| **Strains** | **Strains (abbreviation)** |  |  |
| Escherichia coli TOP10 |  | Invitrogen |  |
| Pichia pastoris GS115 |  | Invitrogen |  |
| GS115/pZACH-(phy)6 | 6 phy | For secreted expression of Phy using 6 PHY expression cassette with zeocin-resistance marker excision |  |
| GS115/ pZHKA-mRFP | mRFP | For expression of mRFP |  |
| GS115/pAOX1_d1+201_-sp-pectinase-HKA | 4 pel | For secreted expression of pectinase using 4 pel expression cassette |  |
| GS115/pZACH-(phy)6/ pZAF | 6 phy/AF | For secreted expression of phytase using 6 PHY expression cassette and overexpression of Fhl1p with zeocin expression cassette |  |
| GS115/pAOX1_d1+201_-sp-pectinase/  pZAF | 4 pel/AF | For secreted expression of pectinase using 4 pel expression cassette and overexpression of Fhl1p with zeocin expression cassette |  |
| GS115/ pZHKA-mRFP-ZACH/pBAF | mRFP/AF | For expression of mRFP using mRFP expression cassette and overexpression of Fhl1p with blasticidin expression cassette |  |

**Figure S8**: Comparison of gene regulation patterns from RNA-Seq and RT-PCR. Genes were selected based on their regulatory behaviour.
